# Infectiousness of Sylvatic and Synanthropic Small Rodents Implicates a Multi-host Reservoir of *Leishmania (Viannia) braziliensis*

**DOI:** 10.1371/journal.pntd.0004137

**Published:** 2015-10-08

**Authors:** Maria S. Andrade, Orin Courtenay, Maria E. F. Brito, Francisco G. Carvalho, Ana Waléria S. Carvalho, Fábia Soares, Silvia M. Carvalho, Pietra L. Costa, Ricardo Zampieri, Lucile M. Floeter-Winter, Jeffrey J. Shaw, Sinval P. Brandão-Filho

**Affiliations:** 1 Department of Immunology, Aggeu Magalhães Research Center-Oswaldo Cruz Foundation (FIOCRUZ), Recife, Pernambuco, Brazil; 2 University of Pernambuco, Recife, Pernambuco, Brazil; 3 Warwick Infectious Disease Epidemiology Research (WIDER) and School of Life Sciences, University Warwick, Coventry, United Kingdom; 4 Biosiences Institute, São Paulo University, São Paulo, São Paulo, Brazil; 5 Biomedical Institute, São Paulo University, São Paulo, São Paulo, Brazil; National Institutes of Health, UNITED STATES

## Abstract

**Background:**

The possibility that a multi-host wildlife reservoir is responsible for maintaining transmission of *Leishmania* (*Viannia*) *braziliensis* causing human cutaneous and mucocutaneous leishmaniasis is tested by comparative analysis of infection progression and infectiousness to sandflies in rodent host species previously shown to have high natural infection prevalences in both sylvatic or/and peridomestic habitats in close proximity to humans in northeast Brazil.

**Methods:**

The clinical and parasitological outcomes, and infectiousness to sandflies, were observed in 54 colonized animals of three species (18 *Necromys lasiurus*, 18 *Nectomys squamipes* and 18 *Rattus rattus*) experimentally infected with high (5.5×10^6^/ml) or low (2.8×10^5^/ml) dose *L*. *(V*.*) braziliensis* (MBOL/BR/2000/CPqAM95) inoculum. Clinical signs of infection were monitored daily. Whole animal xenodiagnoses were performed 6 months post inoculation using *Lutzomyia longipalpis* originating from flies caught in Passira, Pernambuco, after this parasite evaluation was performed at necropsy. Heterogeneities in *Leishmania* parasite loads were measured by quantitative PCR in ear skin, liver and spleen tissues.

**Results:**

All three rodent species proved to establish infection characterized by short-term self-resolving skin lesions, located on ears and tail but not on footpads (one site of inoculation), and variable parasite loads detected in all three tissues with maximum burdens of 8.1×10^3^ (skin), 2.8×10^3^ (spleen), and 8.9×10^2^ (liver). All three host species, 18/18 *N*. *lasiurus*, 10/18 *N*. *squamipes* and 6/18 *R*. *rattus*, also proved infectious to sandflies in cross-sectional study. *R*. *rattus* supported significantly lower tissue parasite loads compared to those in *N*. *lasiurus* and *N*. *squamipes*, and *N*. *lasiurus* appeared to be more infectious, on average, than either *N*. *squamipes* or *R*. *rattus*.

**Conclusions:**

A multi-host reservoir of cutaneous leishmaniasis is indicated in this region of Brazil, though with apparent differences in the competence between the rodent species. The results provide preliminary insights into links between sylvatic and peri-domestic transmission cycles associated with overlaps in the rodent species’ ecological niches.

## Author Summary

Across the Americas, *Leishmania* (*V*.) *braziliensis* is the predominant *Leishmania* species causing cutaneous and mucocutaneous leishmaniasis in humans. Transmitted by Phlebotomine sandflies, questions remain about the epidemiological contributions of the numerous zoonotic and more domestic host species. Domestication of the principal vector and human infection patterns suggest that human infection risk is predominantly peridomestic, whereas control strategies will be more complex if there is a link to a wildlife transmission cycle. Almost no studies have been conducted on the transmission potential of natural hosts of *L*. (*V*.) *braziliensis*. This study evaluates the infectiousness of experimentally infected natural rodent host species, that in different ecological habitats are proposed to act as a single or a multi-host reservoir. Clinical and parasitological development, and the ability to transmit *Leishmania* to sandflies, was observed under experimental conditions using a single strain of *L*. *(V*.) *braziliensis* isolated from the wild rat, *Necromys lasiurus*. Xenodiagnoses were performed with laboratory bred sand fly females established from a local population of *Lutzomyia longipalpis*. All three rodent species developed disseminated subclinical parasitological infections, but clinical signs (lesions) were transient and self-resolving. *N*. *squamipes*, *N*. *lasiurus* and *R*. *rattus* were all infectious when asymptomatic, though their competence in transmission potential appears to differ with *R*. *rattus* showing signs of lower susceptibility. These results provide further evidence that a multi-host reservoir is responsible for maintaining transmission with a bridge between infectious sylvatic and peridomestic rodent populations.

## Introduction

Transmission of zoonotic pathogens may involve one, or typically more than one, reservoir host. Compared to pathogens with single reservoir hosts, those involving multi-host communities usually show reduced transmission rates through a process of zooprophylaxis or “dilution effect” due to heterogeneities in their competence to support pathogen replication and in their infectious duration, resulting in reduced pathogen-host contact, or vector-infectious host contact in the case of vector-borne pathogens [1, 2, 3]. The less common case in nature is that multi-host communities are more homogeneous as competent reservoirs, such that transmission is amplified, otherwise known as zoopotentiation; complexities in these scenarios are discussed elsewhere [2, 4]. Quantification of host heterogeneity has led to a better understanding of transmission dynamics [1, 5, 6], and improved mathematical predictions of transmission hotspots towards development of disease surveillance and control strategies [7, 8].

Zoonotic cutaneous leishmaniasis (ZCL) is a prime example where infection has been detected in multiple host species in different habitats, but where the competence of hosts and sand fly vectors in putative transmission cycles, are not well defined. Across the Americas, the predominant aetiological agent of ZCL is *L*. (*Viannia*.) *braziliensis* causing, in humans, small simple self-healing cutaneous lesions to disfiguring and destructive lesions known as espundia or mucosal leishmaniasis that can result in irreversible disfigurement of the upper nasal tract. In Brazil the dominant parasite causing cutaneous leishmaniasis is *L*. (*V*.) *braziliensis* and there are approximately 26,000 reported new human cases per year but estimates of annual incidences range from 72,800 to 119,600 [9]. *L*. (*V*.) *braziliensis* infections have been identified in sylvatic vectors and small mammals in the Atlantic rainforest biome [10, 11], however transmission has expanded into anthropogenic habitats where infection is observed in more synanthropic and peridomestic species including rodents, marsupials, domestic dogs and equids [11, 12, 13] that may or may not be epidemiologically significant for transmission to humans. Human transmission is predominantly peridomestic as indicated by case age distributions e.g. not limited to adults, forest or plantation workers [14], and the known vector *Lu*. *whitmani*, is captured in large numbers in animals sheds [11, 15]. Control of human ZCL currently relies on human case detection and treatment, however since humans are not thought to be particularly infectious, interrupting transmission necessarily relies on reservoir or/and vector control. There are no comparative transmission studies of *L*. (*V*.) *braziliensis* in small mammal that are indicated as being natural hosts.

By experimental infection, this study aims to compare the reservoir competence of wild and synanthropic rodents previously implicated as reservoirs of *L*. (*V*.) *braziliensis* in northeast Brazil 25. These experiments provide the initial data towards defining their individual *vs* collective susceptibility to infection, ability to support parasite replication, and their infectiousness to phlebotomine sand flies for onward transmission.

## Materials and Methods

Three rodent species (*Necromys lasiurus* syn. *Bolomys lasiurus* (Lund, 1840), *Nectomys squamipes* Brants, 1827 and *Rattus rattus* Linnaeus, 1758) were selected for comparative study as potentially important reservoirs based on demonstrating high prevalences of natural infection or/and high population densities, in previous field studies in endemic ZCL foci in Pernambuco, northeast Brazil [10, 11]. In this region, the reported incidence is 18.5 human cases per 100,000 inhabitants [16]. *N*. *lasiurus* and *N*. *squamipes* are usually associated with Atlantic rain forest and scrub/plantation habitats, whereas *R*. *rattus* is predominantly captured inside houses and animal sheds [11].

Rodent colonies were established at Fiocruz-PE from adult animals live-captured in a well studied foci, Raiz de Dentro, in the Municipality of Amaraji, Pernambuco, northeast Brazil (8° 23’S, 35° 27’W), and identified based on morphological and morphometric characters [17]. A total of 60 F_1_ generation 35–45 day old animals (20 *R*. *rattus*, 20 *N*. *lasiurus* and 20 *N*. *squamipes*), were selected and divided into two groups of 10 animals per species for experimental infection with a high or low dose inoculation of *L*. (*V*.) *braziliensis*, as described below. A control group of two hamsters (*Mesocricetus auratus*) per group was included to confirm the infectivity of the inoculation cultures.

The *L*. (*V*.) *braziliensis* strain (MBOL/BR/2000/CPqAM95) used throughout our experiments was isolated on 08/06/2000 from a *N*. *lasiurus* captured in the Amaraji region, and identified as belonging to zymodeme IOC-Z74, variant 4 and serodeme 1 [18]. This same zymodeme has been isolated from man in the endemic area of Amaraji It was cryopreserved after isolation and then ampoules were thawed and contents passaged from 3 to 4 times before inoculation. In culture and hamsters this strain behaves similarly to other strains of this parasite isolated from other wild rodents and man. Promastigotes were grown in biphasic medium of Blood Agar Base (Difco 45) [19] and Schneider's medium enriched with 20% fetal bovine serum and maintained at 26°C. For experimental infections, the inoculum was prepared from a 7 day old log phase culture containing 2.8×10^5^/ml (low dose) or 5.5×10^6^/ml (high dose) final concentrations according to the doses used for infecting mice with different *Leishmania* species[20]. 7 day old cultures are the inter phase between the log and stationary phases and are composed of infectious metacyclic promastigotes and non-infectious procyclic promastigotes. Twenty replicate animals of each species, *N*. *lasiurus*, *N*. *squamipes* and *R*. *rattus*, were inoculated experimentally with either high (n = 10) or low dose (n = 10) *L*. (*V*.) *braziliensis* Each animal was inoculated in the following sites: left hind paw (0.025 ml), left ear (0.025 mL), and intraperitoneal space (0.05 mL) following a protocol used for experimentally infecting *Proechimys*[21]. Control groups of hamsters (*Mesocricetus auratus*) were inoculated under the same conditions. Animals were followed up for 180 days post inoculation when submitted to xenodiagnoses, and then sacrificed as described below.

### Clinical analysis

After experimental infection, animals were monitored daily to detect any clinical changes including lesions on the inoculation site, hair loss, or splenomegaly.

### Xenodiagnosis

Xenodiagnosis was performed on 18 of each rodent species six months after inoculation using 7-day old sand flies, from the first generation *Lu*. *longipalpis* captured in a well studied foci, in the Municipality of Passira, Pernambuco, northeast Brazil (7° 56’S, 35° 35’W). The mating song of this population has been determined as a burst type, being very similar to Camara and Bacarena populations of Pará State [22]. Burst song populations are principally coastal and all have the cembrene-1 pheromone[23]. The animals were anesthetized with ketamine hydrochloride at 10% and placed in cages into which female sand flies were released and allowed to feed for about 40 minutes in the presence of a similar number of male sand flies in order to induce feeding and copulation. Blood-fed females were then transferred to plastic pots that were stored in boxes with light filter protection and kept under controlled laboratory conditions until the seventh day when they were dissected to detect promastigote forms under optical microscopy.

### Necropsy and parasitology procedures

After conclusion of xenodiagnosis, the animals were euthanized with a CO^2^ inhalation process. Fragments of approximately 50mg of ear skin, spleen and liver were collected from each euthanized animals, and *Leishmania* parasite DNA quantified by quantitative PCR (qPCR).

### DNA extraction and molecular detection of parasites by qPCR

DNA was extracted from tissues using DNeasy Blood & Tissue kit (Qiagen) according to the manufacturer’s protocol. The initial molecular detection protocol consisted of a nested PCR assay using two pairs of SSU rDNA (Small Subunit Ribosomal gene) derived oligonucleotides. The first PCR used SSU rDNA primers [24] that amplify a conserved region of all trypanosomatids (S12: 5’-GGTTGATTCCGTCAACGGAC-3’ and S4: 5’-GATCCAGCTGCAGGTTCACC-3’); internal oligonucleotides PCR products were analyzed by electrophoresis in agarose gel. The second reaction was a real time PCR (qPCR) to quantify the parasite load [25] using primers that amplify a common region of the *Leishmania (Viannia)* subgenus (S17: 5’-CCAAGCTGCCCAGTAGAAT-3’ and S18: 5’-TCGGGCGGATAAAACACC-3’). The quantification protocol consisted of a real time SYBR-Green PCR; tissue parasite loads were standardized as number of SSU rDNA copies per host glyceraldehyde-3-phosphate dehydrogenase (GAPDH) copy number. The PCR conditions were optimized to generate a single melting curve of the product.

### Data analysis

Established experimental infection was defined as the presence of one or more condition: development of skin lesions associated with symptomatic rodent ZCL, detection of splenomegaly at necropsy, qPCR detection of *Leishmania* in tissue samples (ear skin, liver, spleen), or infectiousness to sand flies. For statistical analyses, *Leishmania* loads were log_10_+1 transformed and tested using general linearised Poisson models (negative binomial over-dispersion coefficient α<0.088, χ^2^<0.94, P>0.281 in each case). The relationships between infectiousness (proportion of sandflies infected) or presence/absence of skin lesions against independent variables were analysed using logistic regression weighted by sample sizes. Depending on the outcome of interest, multivariate analysis adjusted for covariates including inoculum size (high dose or low dose), skin tissue log_10_ parasite load, inoculum size × skin log_10_ load interaction term, times to lesion onset and lesion recovery, and rodent species. All analyses were carried out using Stata v.13.1 software (Stata Corporation, College Station, Texas, USA).

### Ethical and safety considerations

Approvals to conduct this study and to capture wild animals to establish laboratory colonies were obtained from the Animal Research Ethics Committee of Oswaldo Cruz Foundation, Rio de Janeiro (Protocol No. L-056/05), and endorsed by the Brazilian Institute of Environment (IBAMA License No. 12.749–1). All the experimental animals were handled in accordance with the recommended guidelines and safety measures;. captured animals, experimental animals and the colonies were all kept in quarantine that involved microbiological testing, safety barriers with micro- and macro-isolators, and under strict hygiene conditions [26, 27] following security standards (International Organization for Standardization—IS0/15189).

## Results

### Infection

Within 3 months of being inoculated and before sampling four high dose rodents (2 *N*. *lasiurus*, 2 *N*. *squamipes*) and one low dose *R*. *rattus* died, thus final follow-up sample sizes were therefore 18 *N*. *lasiurus*, 18 *N*. *squamipes* and 19 *R*. *rattus* (55 animals in total).

Infections were confirmed in 18/18 *N*. *lasiurus*, 18/18 *N*. *squamipes* and 9/19 *R*. *rattus* by molecular methods and xenodiagnosis (Tables 1 & 2). The two experimental inoculum doses appeared similar in successfully establishing rodent infection (26/29 high dose vs 19/26 low dose animals) (χ^2^ = 2.55 P = 0.614), though some specific differences were observed as described below. All control hamsters developed infection, that included lesions at the inoculation site and all tissues were positive by nested PCR that confirmed the infectiousness of the high and low dose innoculum.

**Table 1:** Proportion of rodents experimentally inoculated with high or low dose *L*. *braziliensis* that showed signs of established infection defined as presentation of skin lesions, spenomegaly at necropsy, detection of *Leishmania* in tissue samples, or/and xenopositivity.

Experimental inoculation	*Leishmania* detected in tissues^1^	Presence of skin lesions	Spleno-megaly at necropsy	Infectious to sand flies^2^	animals infected/ inoculated^3^
High dose					
*Necromys lasiurus*	7/8	8*/8	3/8	8/8	8/8
*Nectomys squamipes*	6/8	3/8	0/8	1/8	8/8
*Rattus rattus*	4/10	2/10	0/10	0/9	3/10
subtotal	14/26	13/26	3/26	9/25	19/26
Low dose					
*Necromys lasiurus*	9/10	0/10	1/10	10/10	10/10
*Nectomys squamipes*	10/10	0/10	2/10	9/10	10/10
*Rattus rattus*	2/9	1*/9	0/9	6/9	6/9
subtotal	21/29	1/29	3/29	25/29	26/29

1 Ear skin, liver and spleen tissue samples screened for *Leishmania* by qPCR.

2 Infectiousness defined as infecting ≥1 dissected sand fly by xenodiagnosis.

3 Total number of experimentally inoculated animals that survived to follow-up

* Metastatic lesions at base of tail—7 *N*.*lasiurus* (4 had ear lesion) and 1 *R*.*rattus*.

**Table 2:** Crude proportions of blood-fed dissected female sand flies that were infected by rodents experimentally inoculated with high or low dose *L*. *braziliensis*.

Experimental inoculation dose	N animals xenopositive/tested (n blood-fed sand flies dissected)	Median proportion of flies infected per rodent binomial 95% C.I.s
High dose		
*Necromys lasiurus*	8/8 (77)	0.42 0.115–0.538
*Nectomys squamipes*	1/8 (60)	0.00 0.00–0.036
*Rattus rattus*	0/9 (162)	0.00 0.00–0.00
Low dose		
*Necromys lasiurus*	10/10 (206)	0.36 0.240–0.443
*Nectomys squamipes*	9/10 (141)	0.35 0.129–0.406
*Rattus rattus*	6/9 (101)	0.29 0.00–0.416

### Clinical outcomes

**Fig 1:**
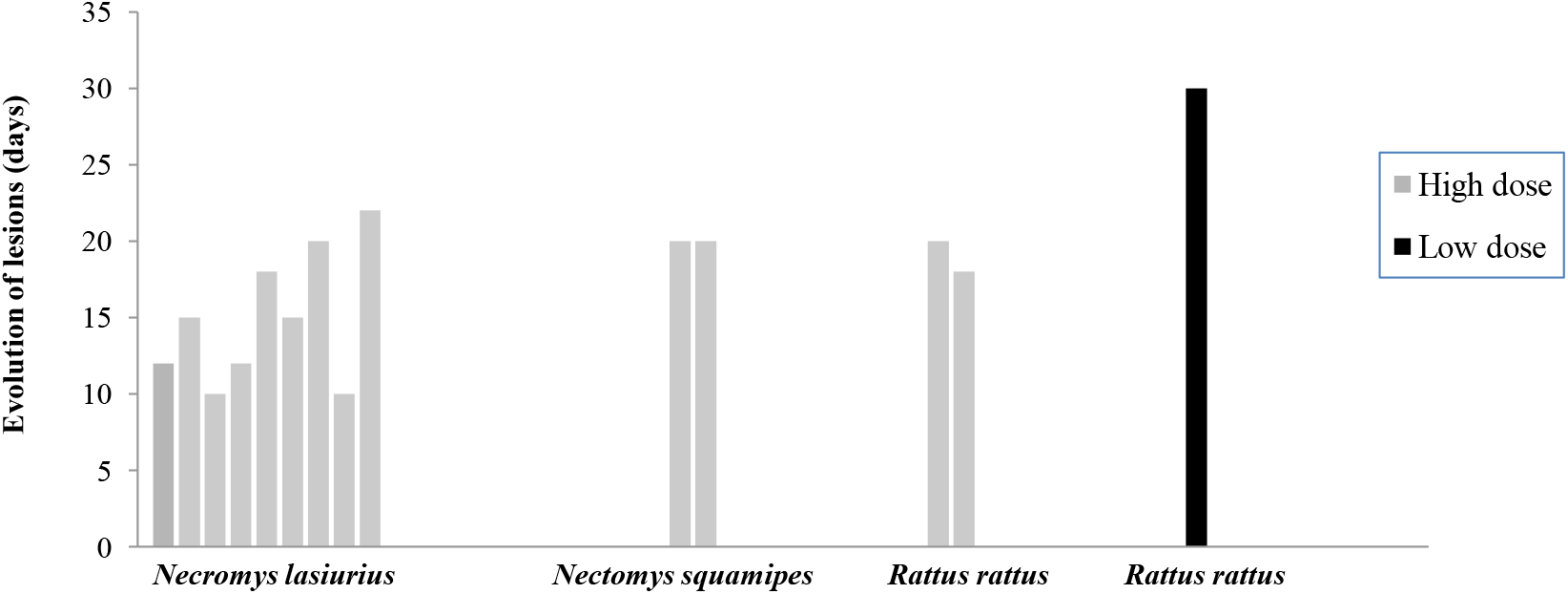
Bar graph showing the duration of skin lesions in days of laboratory bred *Necromys lasiurus*, *Nectomys squamipes* and *Rattus rattus* inoculated with different concentrations (High dose = 5.5×10^6^/ml and Low dose = 2.8×10^5^/ml) of *Leishmania (Viannia*) *braziliensis (*strain (MBOL/BR/2000/CPqAM95).

One or more skin lesions associated with infection were observed in 14/55 animals, where 9 and 4 of the high dose animals respectively developed 1 and 2 lesions located on the ear (1 *N*. *lasiurus*, 3 *N*. *squamipes*, 2 *R*.*rattus*), ear and tail base (4 *N*. *lasiurus*) tail base (3 *N*. *lasiurus*) and one low dose animal developed a tail base lesion (1 *R*.*rattus*); no lesions were observed on the footpads at the site of experimental inoculation in any animal. The higher experimental dose induced a higher proportion of animals to present skin lesions (13/26 50%) compared to the low dose group (1/29 3.5%) (χ^2^ = 9.44 P = 0.002) (Table 1 and Fig 1). Splenomegaly at necropsy was rare for both doses (Table 1). At the time that the xenodiagnoses were performed no animals had visible lesions.

In high dose animals, average times to lesion development post inoculation were 38 (95% CI: 33.9–42.6), 48 (33.3–62.0) and 51 (51.0–51.0) days respectively for *N*. *lasiurus*, *N*. *squamipes* and *R*. *rattus*. Time to lesion onset was statistically shorter in *N*. *lasiurus* than *N*. *squamipes* or *R*. *rattus* (z>2.88, P< = 0.004), but not dissimilar between *N*. *squamipes* and *R*. *rattus* (z = 0.85, P = 0.398). All lesions spontaneously recovered within one month of onset, after an average 14 (95% CI: 10.9–17.1), 21 (17.8–23.5) and 19 (6.3–31.7) days for the three species, respectively. Lesion duration was shorter (i.e. faster recovery) in *N*. *lasiurus* compared to *N*. *squamipes* or to *R*. *rattus* (z>2.01, P< = 0.044), but not statistically different between *N*. *squamipes* and *R*. *rattus* (z = -0.58, P = 0.561).

### Parasitology

**Fig 2:**
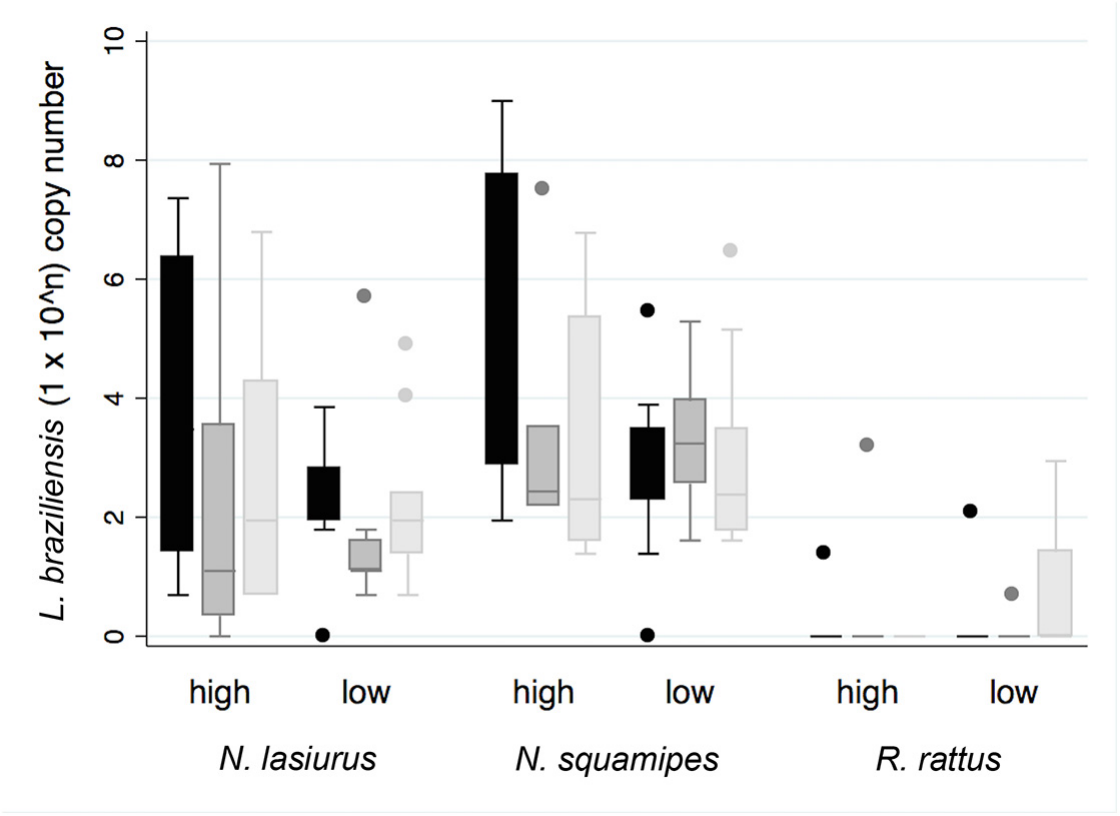
*L*. *braziliensis* log_10_+1 cell counts estimated by qPCR in tissue samples collected from rodent species that received initial high or low inoculum dose (x-axis). Colour shades represent tissues: skin (black), spleen (dark grey), and liver (light grey). Cell counts were standardised to the number of host gene GAPDH copies.

The results of tissue parasite loads were quantified by qPCR in single skin, spleen, and liver tissue samples from all follow-up animals at necropsy are show in Table 3. Maximum tissue burdens were 8.1×10^3^ in skin, 2.8×10^3^ in spleen, and 8.9×10^2^ in liver samples. Substantial variation in *Leishmania* loads were observed between individual tissues, animals, and inocula dose (Table 3 and Fig 2). Log_10_ parasite loads in the three tissues were only moderately correlated (Spearman’s r = 0.64–0.67, P<0.001). Average log_10_ skin tissue loads were lower in low dose *vs* high dose animals (z = -2.87, P = 0.004), whereas the variation in liver and spleen tissues loads did not significantly differ between dose groups when adjusting for inter-species variation (z<0.84, P>0.05). *Leishmania* loads in *R*. *rattus* tissues were generally lower compared to those in *N*. *lasiurus* (all tissue comparisons: z>-4.1, P<0.0001) or in *N*. *squamipes* (z>-4.9, P<0.0001), whereas those of *N*. *lasiurus* and *N*. *squamipes* were not significantly different from one another (z<2.4, NS).

**Table 3:** *L*. *braziliensis* parasite loads^1^ detected in tissue samples from rodents with infections established by high or low dose initial inoculum.

Rodent/dose	Tissue sample	Pos/N tested	mean	Median (IQR)^2^	range
High dose					
*N*. *lasiurus*	skin	8/8	3.6×10^2^	52 (2–1006)	1–1.6×10^3^
	spleen	6/8	3.9×10^2^	2 (0–1110)	0–2.8×10^3^
	liver	7/7	1.4×10^2^	6 (6–632)	1–8.9×10^2^
*N*. *squamipes*	skin	7/7	1.5×10^3^	56 (10–6263)	6–8.1×10^3^
	Spleen	6/6	3.2×10^2^	11 (8–1641)	1.8×10^3^
	liver	7/7	1.6×10^2^	9 (3–668)	3–8.8×10^2^
*R*. *rattus*	skin	1/9	0.3	0 (0–0)	0–3
	spleen	1/9	2.6	0 (0–0)	0–2.3×10^1^
	liver	0/8	0	0 (0–0)	0–0
Low dose					
*N*. *lasiurus*	skin	8/9	1.3×10^1^	8 (5–17)	0–4.6×10^1^
	spleen	9/9	3.4×10^1^	2 (2–5)	1–2.9×10^2^
	liver	9/9	2.4×10^1^	6 (2–51	1–1.3×10^2^
*N*. *squamipes*	skin	9/10	3.7×10^1^	14 (5–43)	0–2.3×10^2^
	spleen	10/10	4.4×10^1^	25 (9–61)	4–2.0×10^2^
	liver	10/10	9.0×10^1^	10 (4–127)	4–6.4×10^2^
*R*. *rattus*	skin	1/8	0.9	0 (0–2)	0–7
	spleen	1/9	0.1	0 (0–0)	0–1
	liver	2/8	4.3	0 (0–17)	0–1.8×10^1^

1 *Leishmania* copy numbers (loads) measured by qPCR were standardized to the number of host gene GAPDH copies.

2 IQR interquartile range

### Xenodiagnosis

Six months after being infected a total of 299 and 448 female *Lu*. *longipalpis* fed respectively on 25 high and 29 low dose animals. No lesions were present on any animals at this time. Flagellates were detected in sand flies fed on 18/18 *N*. *lasiurus*, 10/18 *N*. *squamipes* and 6/18 *R*. *rattus*. The low dose inoculum tended to induce a higher proportion of animals to be infectiousness to sandflies (25/29 86.2%) compared to the high dose group (9/25 36%) (χ^2^ = 3.46 P = 0.063) (Table 2). A median 15 (95% C.I.: 6.9–24.1), 12 (4.3–14.7), and 17 (6.6–21.0) engorged females sand flies fed on *N*. *lasiurus*, *N*. *squamipes* and *R*. *rattus*, survived to dissection.

Mutlivariate logistic regression of the proportion of flies infected (for N = 34 animals), adjusting for skin tissue *Leishmania* load × inoculum group interactions, indicated that *N*. *lasiurus* tended to be more infectious on average than either *N*. *squamipes* (z = -2.91, P = 0.004) or *R*. *rattus* (z = -1.73, P = 0.084). The infectiousness of *N*. *squamipes* and *R*. *rattus* was not statstically different from each other (z = 0.43, P = 0.670). Despite the general low tissue parasite loads in *R*. *rattus*, a significant proportion of low dose animals were infectious to sandflies (Table 2). Notwithstanding, the proportion of exposed sand flies infected was generally positively associated with the log_10_ parasite loads in skin tissue when accounting for differences between rodent species (z = 4.69, P<0.001), but was not associated with either time to lesion onset post inoculation (z-1.56, NS) or to lesion duration (z = 0.51, NS).

## Discussion

This study investigated the comparative development of experimental infection in three putative rodent reservoir species, and their relative ability to transmit *L*. *braziliensis* to blood-feeding sandflies. We show that all three rodent species established infections, supported persistent *Leishmania* burdens in multiple tissue, and presented transient clinical lesions which developed within an average 38–51 days post inoculation, that spontaneously resolved within an average 14–19 days. All three rodent species were also able to infect sandflies as demonstrated by xenodiagnosis performed at c. 6 months post inoculation, by which time all skin lesions had visually healed. We found an association between infectiousness to sand flies and *Leishmania* loads in ear skin tissue, but not to lesion presence/absence, onset or duration time. Comparing rodent species, *N*. *lasiurus* tended to have a greater likelihood of being infectious (18/18 animals), compared to the other two species, and for comparable skin log_10_ *Leishmania* loads, both *N*. *lasiurus* and *N*. *squamipes* infected a greater average proportion of sand flies than did *R*. *rattus*. *R*. *rattus* also appeared less likely to establish experimental infection at either inoculum dose, evidenced by less clinical signs and lower parasite loads. Despite these observations, the low dose group of *R*. *rattus* still proved to be infectious to a small proportion of sand flies, at least at 6 months post inoculation. These collective results suggest that the investigated rodent species represent a multi-host reservoir, though with variable reservoir competence by cross-sectional comparison. Whether any single rodent species can maintain a transmission cycle independently (R_0_>1) requires further study and parameter estimation [28]. For example, there is likely to be a trade-off between duration and degree of infectiousness relative to the host’s life expectancy: low-level infectiousness over sustained periods could be more significant than high-level infectiousness over a shortened life expectancy resulting from acute infection; data on their comparative longitudinal profile to indicate life-long transmission potential would be informative (e.g.[29]).

Our observations of *R*. *rattus* may indicate a greater innate resistance of this species to *L*. (*V*.) *braziliensis* than the other rodent species. It is also possible that this host resolved higher parasitological infections within a shorter time frame than our sampling regime. *R*. *rattus* experimentally infected with another cutaneous causing species, *L*. *tropica*, presented asymptomatic infections despite ear tissue parasite loads of 4×10^3^−10^6^ with no significant decline over 24m follow-up, and were infectious to a low proportion of sandflies (0–7%) even when fed on the site of experimental inoculation [30]. In contrast to current results, a threshold of infectiousness is positively associated with high parasite loads in ear skin of dogs naturally infected with *L*. *infantum* [31]. Unexpectedly, we observed infectiousness to sandflies to be higher in animals inoculated with the low dose compared to the high dose, the latter was associated with skin lesions and higher parasitaemia. In nature *Leishmania* inoculum sizes from a single infected sandfly have been found to be in the order of 4–40,000 metacyclic promastigotes [32, 33, 34]. No such figures are available for *L*. (*V*.) *braziliensis*. Ideally in such experiments the infecting organisms should come from the bite of a sandfly but at the moment this is technically impossible for *L*. (*V*.) *braziliensis*. In the absence of this possibility the inoculum should contain a similar number of organisms to those delivered by the sandfly. We calculate that our lower dose contained a maximum of approximately 22,000 metacyclic promastigotes which is within the higher range of parasites delivered by a sandfly infected with a leishmania of the subgenus *L*. (*Leishmania*), but our higher dose did not fall within the above mentioned range. As we have already said lesions are not at all typical of natural *L*. (*V*.) *braziliensis* infections and because the higher dose produced lesions in many animals we decided to use an inoculum containing fewer organisms in our second experiment. Other factors influence an infection and besides the number of metacyclic promastigotes such sandfly and parasite antigens accompanying the bite and previous exposure to sand fly saliva. The latter may mount a protective response against lesion development, after subsequent challenge [35]. None of our rodents exposed to sand fly bites before the xenodiagnosis nor was any sand fly saliva associated with the inoculation. Significantly more animals, which received the higher dose, had lesions compared to only a single animal that received the lower dose developed a lesion, which mirrored more closely what we have seen in wild infections. The fact that fewer animals that received the higher dose were infectious may reflect a forced immunity produced by more parasites.

Natural infections of *L*. (*V*.) *braziliensis* in free-ranging small mammals are occult [11, 36, 37, 38] in contrast to rodents naturally infected with some *Leishmania* belonging to the subgenus *L*.*(Leishmania)* such as *L*. *(L*.*) amazonensis* and *L*. *(L*.*) major* [39] that often present parasite rich lesions. Since 1996 we have examined over 1,000 small silvatic mammals for infections of *L*. (*V*.) *braziliensis* and periodically isolated this parasite from blood, spleen and liver [11]. The skin from wild animals was positive in PCR tests but we have never managed to isolate the parasite from this tissue nor have we seen any leishmanial skin lesions. It’s quite feasible that flies become infected from parasites present in the skin as well as parasites liberated into the blood from the liver and spleen. *L*. (*V*.) *braziliensis* has also been detected molecularly in apparently normal skin of asymptomatic wild mammals captured in other endemic areas [37, 38] but no isolations were obtained. It is possible that cross-sectional field studies fail to detect short-lived clinical signs in naturally infected captured rodents, a question that can only be resolved by longitudinal follow-up studies. Indeed little is known of the tissue tropism of *Leishmania* species in their natural hosts, and in laboratory animals some strains of *L*. (*V*.) *braziliensis* are predominantly cutaneous while others also visceralize [40].

The appearance of metastatic lesions at the base of the tail in 13 of our high dose animals and 1 of the low dose animals and the complete absence of lesions at the site of inoculation on the foot pad is extremely interesting. The base of the tail is one preferential feeding site for sand flies and leishmanial lesions have been frequently observed at the base of the tail for *L*.(*L*.) *mexicana* and *L*.(*L*.) *amazonensis* [41, 42]. So why did the lesions appear at this site? A possible reason is a tissue tropism which favors the parasite being in a place where it is readily available to the vector and may be present in the absence of visible lesions. This indicates that future studies on reservoirs need to concentrate on material from the tail base irrespective of the presence of a lesion.

We detected a poor correlation between the log_10_ parasite loads in rodent skin, liver and spleen tissues. In longitudinal studies of *Leishmania* loads in dog tissues naturally infected with *L*. *infantum*, we similarly observed a poor correlation between tissue loads, however, this was explained by the observed proportional shift in parasite loads in the skin relative to in bone marrow which increased during the time course of infection [31]. Many of the clinical forms of *L*. (*V*.) *braziliensis* in man, such as disseminated cutaneous and mucocutaneous presentations, are considered due to metastatic spread from an initial active or cured lesion or some internal tissue. *L*. *tropica* loads of 7.5×10^3^–6×10^4^/cm^2^ were reported in cutaneous sites (tail tissue, but not in liver, spleen, blood, or bone marrow) disseminated from the experimental inoculation site in *R*. *rattus* [30]. It appears that *L*. (*V*.) *braziliensis*, and other *Leishmania* species, have adopted a strategy in the host to become persistently available to sandflies in the skin and peripheral blood following parasites dissemination from the site of inoculation or/and multiplication in liver and spleen tissues.

One caveat of the current study is that xenodiagnosis was performed using *Lu*. *longipalpis* rather than *Lu*. *whitmani*, a confirmed vector of *L*. (*V*.) *braziliensis*. *Lu longipalpis* is highly susceptible to many *Leishmania* species, including a number of *L*. (*Viannia*) species [43] thus being classified as a permissive vector [44], and has been considered a potential vector of *L*. (*L*.) *amazonensis* and *L*. (*V*.) *braziliensis* in Brazil [45]. Here we necessarily treat the xenodiagnosis results as comparative values, assuming that any bias associated with relevant vectorial capacity components is uniform across rodent species.

*Lu*. *whitmani* and *Lu*. *intermedia* as well as other sand fly species are considered to be competent vectors of *L*.(*V*.) *braziliensis*, based on epidemiological and parasitological observations of wild caught infected female flies. The absence of suitable models to assess vector competence for *L*.(*V*.) *braziliensis* is reflected by the fact that there is only one published account of the successful experimental transmission and this was with a naturally infected fly [46]. *Lu*. *longipalpis* is considered to be a permissive vector [44] because it supports the development and adherence of different *Leishmania* including species of subgenus *L*.(*Viannia*) [47] [48] as well as other promastigote producing heteroxenous parasites, such as *Endotrypanum* [49]. However, its capacity as a vector involving colonization of the cardial region and the production of metacyclic promastigotes has yet to be assessed for this group of parasites. Within this frame work we consider that it is perfectly valid to use *Lu*. *longipalpis* to assess the comparative infectiousness of these rodent hosts. Whether its sensitivity in detecting infection is equal to that of the natural vectors can only be determined by comparative experiments. *Lu*. *longipalpis* is a complex of sibling species[22] so another question is are there differences in their susceptibility to infection? So far there is no evidence to suggest such differences exist. Our flies belong to the burst song group which is the same as flies that have been widely used by other workers under the name Marajó.

The rodent species evaluated in the current study were selected on the basis of consistent high infection rates or/and high abundance in multiple field studies in northeast Brazil [10, 11]. *N*. *squamipes* is the largest rodent of the three species (c. 240gm *vs R*. *rattus* 50gm and *N*. *lasiurus* 160gm) which may attract relatively more sand flies [15, 50, 51]. The comparative roles of domesticated hosts, such as dogs and equids, have yet to be quantified: it is known that infection prevalences in dogs are comparable to those in rodents [11, 52], and that dogs can infect *Lu*. *whitmani* when fed on their skin lesions [53, 54]. However, there are few xenodiagnosis studies on naturally infected hosts of *Leishmania*, and detection of infection does not necessarily equate to transmission potential (e.g. [55]).

This study provides some preliminary insights into the likely transition from the assumed original transmission cycle of *L*. (*V*.) *braziliensis* involving Atlantic forest small mammals and sand fly vectors, to a more peridomestic cycle involving, not least, the infectious rodents described here, that are associated with overlapping sylvatic and peridomestic habitats (*N*. *lasiurus* and *N*. *squamipes*) and domestic habitats (*R*. *rattus*) respectively [11, 15]. The expansion of this apparent “bridge” between sylvatic and peridomestic transmission habitats are facilitated by the widespread deforestation and conversion of remaining forest to sugarcane and banana plantations. The consequence of environmental shifts on multi-host identity and diversity e.g. proportion of opportunistic and/or competent host species in anthropogenic habitats, may prove to be positive or negative for human transmission [2]. Potential changes in zoopotentiation or zooprophylaxis may be offset by the sand fly vector’s restricted feeding behaviour: *Lu*. *whitmani* demonstrates a degree of domesticity, feeding site and host choice loyalty, potentially limiting vector-host contact to more predominant competent species [15, 51, 56]. This focus lies at the southern edge of the geographical range of *N*. *squamipes* [57], with the possibility that other species inhabit it’s ecological niche elsewhere [10]. Research is now needed to place the current results in context of longitudinal field studies of natural infection and transmission and including in domesticated animal hosts.
